# An update of skin permeability data based on a systematic review of recent research

**DOI:** 10.1038/s41597-024-03026-4

**Published:** 2024-02-21

**Authors:** Lisa Chedik, Shamkhal Baybekov, Frédéric Cosnier, Gilles Marcou, Alexandre Varnek, Catherine Champmartin

**Affiliations:** 1 Institut national de recherche et de sécurité pour la prévention des accidents du travail et des maladies professionnelles (INRS), Dept Toxicologie et Biométrologie, 1 rue du Morvan, 54519 Vandoeuvre-lès-Nancy, France; 2 Laboratoire de Chémoinformatique UMR 7140 CNRS, Institut Le Bel, University of Strasbourg, 4 Rue Blaise Pascal, 67081 Strasbourg, France

**Keywords:** Data integration, Occupational health, Drug delivery

## Abstract

The cutaneous absorption parameters of xenobiotics are crucial for the development of drugs and cosmetics, as well as for assessing environmental and occupational chemical risks. Despite the great variability in the design of experimental conditions due to uncertain international guidelines, datasets like HuskinDB have been created to report skin absorption endpoints. This review updates available skin permeability data by rigorously compiling research published between 2012 and 2021. Inclusion and exclusion criteria have been selected to build the most harmonized and reusable dataset possible. The Generative Topographic Mapping method was applied to the present dataset and compared to HuskinDB to monitor the progress in skin permeability research and locate chemotypes of particular concern. The open-source dataset (SkinPiX) includes steady-state flux, maximum flux, lag time and permeability coefficient results for the substances tested, as well as relevant information on experimental parameters that can impact the data. It can be used to extract subsets of data for comparisons and to build predictive models.

## Background & Summary

The skin plays an important protective role against external aggression, thanks mainly to the properties of its outermost layer: the *stratum corneum* (SC). However, the skin is not an absolute barrier and xenobiotics can penetrate the *stratum corneum*, diffuse into the viable epidermis and enter the general circulation through the capillaries of the dermis. The different steps of the transport process have been described elsewhere^1^.

Accurate assessment of the rate and extent of the percutaneous absorption of xenobiotics is of paramount importance for the development of new pharmaceutical and cosmetic products applied to the skin to ensure or prevent their absorption into the deep layers of the skin. These data are also necessary to assess the chemical risk of substances when cutaneous environmental or occupational exposure exists.

These substances deposited on the skin can indeed be responsible for irritation, sensitizing effects or general toxic effects and require *ad hoc* regulatory labeling. For instance, the REACH Annex VII mentions skin sensitization, irritation and corrosion assessments for substances produced and imported into Europe in volumes above one ton. In addition, the dermal route of exposure must be addressed in Annex VI [Regulation (EC) No 1907/2006 of the European Parliament and of the Council of 18 December 2006 concerning the Registration, Evaluation, Authorization and Restriction of Chemicals (REACH), establishing a European Chemicals Agency, amending Directive 1999/45/EC and repealing Council Regulation (EEC) No 793/93 and Commission Regulation (EC) No 1488/94 as well as Council Directive 76/769/EEC and Commission Directives 91/155/EEC, 93/67/EEC, 93/105/EC and 2000/21/EC].

**Fig. 1:**
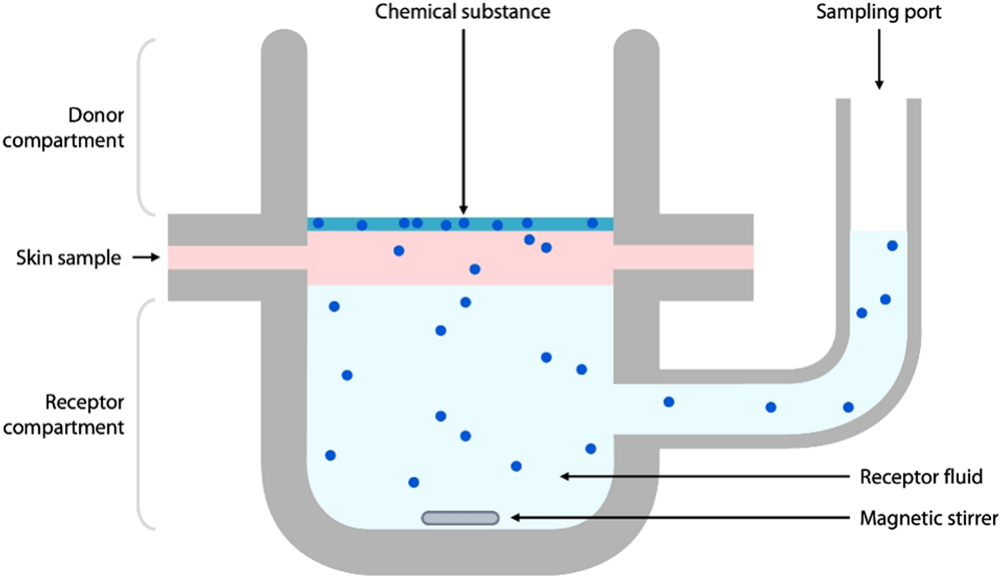
Schematic illustration of a static Franz diffusion cell.

In practice, the permeation of a chemical substance through the skin, assimilated with a passive diffusion phenomenon, can be studied experimentally *in vitro* using a diffusion cell device composed of donor and acceptor compartments between which the skin (*stratum corneum* side up) is placed (Fig. 1).

These experiments measure the quantity of chemical (Q in µg) passing through the skin barrier per unit of skin surface (S in cm^2^).

**Fig. 2:**
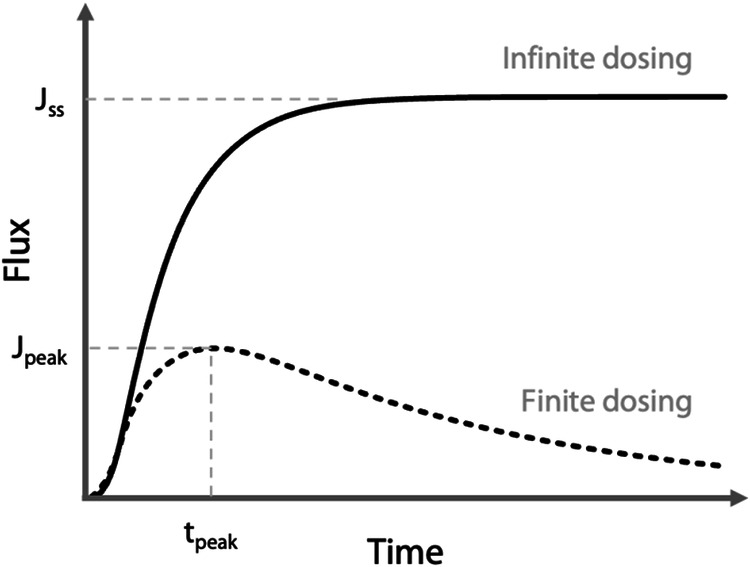
Theoretical change of outgoing flux for infinite (solid line) and finite dosing (dashed line). J_peak_, t_peak_, J_ss_ correspond to the maximum flux, the time of the maximum flux and the steady-state flux, respectively.

The experiments of percutaneous absorption can be conducted in finite dose conditions, i.e. a “finite” quantity of the chemical is applied to the skin so that a maximum flux (noted J_peak_) of the test substance is achieved during a certain time interval (t_peak_) but is not maintained (Fig. 2). This contrasts with experiments with infinite doses where the concentration of the chemical in the donor compartment remains relatively constant throughout the experiment, ensuring the attainment and sustained maintenance of a steady-state flux J_ss_.

Using these infinite dose conditions and the steady-state flux data, it is possible to calculate the permeability coefficient, K_p_ with the following equation:

$${J}_{ss}={K}_{p}\times \Delta {{\rm{C}}}_{{\rm{s}}}$$

J_ss_ is the steady-state chemical transfer rate per unit area (µg•cm^−2^•h^−1^). Note that, when the substance is applied in pure form (neat liquid) or at its saturated concentration, the steady-state flux is called J_max_.

∆C_s_ is the difference in the concentration (µg•cm^−3^) of the chemical diffused between the inlet and outlet of the skin. Given the definition of infinite dose, ∆C_s_ is often approximated by the concentration of the chemical in the donor compartment at the beginning of the experiment (C_0_).

K_p_, the permeability coefficient (cm•h^−1^), reflects the ability of a membrane to let a substance permeate through it.

**Fig. 3:**
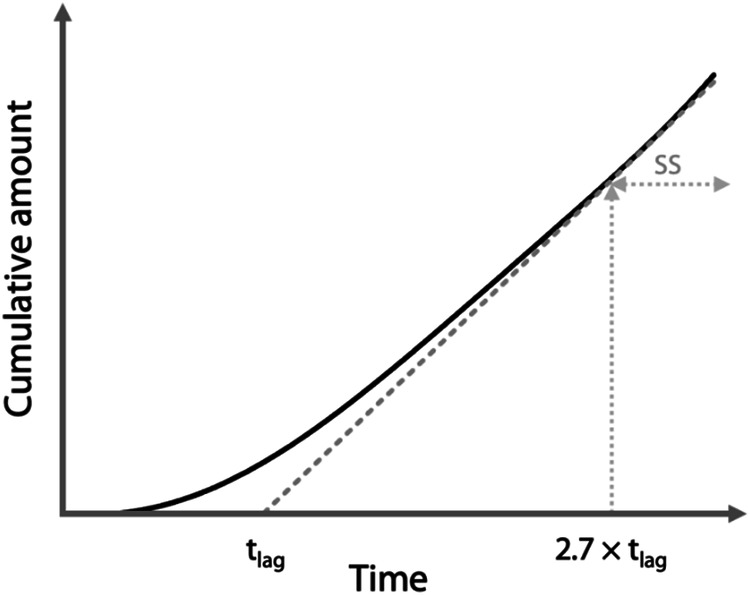
Cumulative amount of the tested chemical over time in the acceptor compartment during an infinite dose experiment. The solid line represents the whole experiment, and the dashed line represents the extrapolation of the linear steady-state phase (SS). The intersection of the dashed line with the time axis is the lag time (t_lag_).

The amount of compound in the acceptor compartment increases exponentially over time until reaching the steady-state. J_ss_ is typically obtained from the slope of the linear part of the curve. The intersection of the linearized steady-state phase and time axis denotes the lag time, t_lag_ (Fig. 3). The t_lag_ reflects the time it takes for the substance to cross the skin barrier.

Despite the fact that the first publications on the *in vitro* percutaneous absorption of xenobiotics date back to the 1960s, this research topic has not been studied extensively. Over the past 30 years, efforts have been made and initiatives taken to aggregate the available data on the skin permeation of xenobiotics. In 1990, Flynn collected human skin permeability coefficient data for the first time *in vitro* for over 90 chemicals^2^. Then, the EDETOX database^3^ reported *in vivo* and *in vitro* literature data obtained for different species in a free databank which is still available on the web at http://edetox.ncl.ac.uk (updated in 2016). Samaras *et al*., extracted the *in vitro* human dataset from EDETOX and completed it with data obtained between 2001 and 2010^4^. This dataset is freely available for consultation only as a spreadsheet in the supplementary data. Finally, HuskinDB lists all the percutaneous absorption data from *in vitro* studies on human skin until 2011^5^. The corresponding database is freely accessible on https://huskindb.drug-design.de or 10.7303/syn21998881, (last access 12/04/2023). Although this database represents a step forward compared to the two previous ones because it provides a better description of experimental conditions, it reports data on only 253 substances and as for the previous databases the inclusion/exclusion criteria conditions deserve to be more extensively described. It should be noted that in the publications selected for these different databases, not all the experimental conditions are systematically reported.

Cheruvu *et al*.^6^ recently proposed an update to these data stemmed from a review paper^7^. The authors focused on maximal flux (J_max_), and permeability coefficient (K_p_) values collected from *in vitro* human skin permeation tests performed on human epidermal membranes or isolated stratum corneum at infinite dosing but the use of this latter type of skin can be debated. They also reported physicochemical properties and experimental conditions under which the data was generated (temperature, skin thickness, and skin integrity). Other parameters important for percutaneous absorption should have been reported (e.g. skin donor source, skin preparation techniques, skin source, storage duration and temperature, donor and acceptor pH, cell type).

The lack of data in the field of percutaneous absorption is particularly problematic for the generation of efficient predictive models on skin permeation such as QSPR (Quantitative Structure-Permeability Relationship) models. This implies that most existing *in silico* models are trained on the Flynn dataset^2^ or variations of it, and have very limited domains of applicability.

In addition, the comparison of data between different publications can be tricky because, although international guidelines (OECD Guidance Document 28 (GD28) for conducting skin absorption studies^8^, Test Guideline 428 (TG428) for measuring skin absorption of chemicals *in vitro*^9^, and the OECD Guidance Notes 156 (GN156) on dermal absorption issued in 2019^10^ and 2022^11^) give recommendations on experimental conditions and set-ups, they remain relatively imprecise and leave room for many variations in experimental designs that are left to the discretion of the experimenter. Many of these factors have a significant influence on the results of percutaneous absorption experiments^12^, such as the donor type, also called vehicle^13,14^, the skin donor type, the skin source site, the layer used and the experimental cell device (see usage notes). These guidelines have been written to be broadly applicable to many skin exposure contexts, hence this variability is unavoidable. As mentioned by others^15–17^, a better defined standard protocol would be desirable for comparison between studies and laboratories, knowledge aggregation and rational decision making.

Here we present SkinPiX (Skin Permeation of identified Xenobiotics), a new dataset obtained after the systematic collection of the available literature on human percutaneous absorption published after 2012. The dataset contains flux, t_lag_ and K_p_ data of the substances studied but also information specifying the experimental conditions. The scientific literature was curated manually by scientists from INRS (Reference body for occupational risk prevention in France), experts in percutaneous absorption. Exclusion or inclusion criteria were applied as explained in the Methods section.

When the information is available in the publication, SkinPiX indicates, in addition to the percutaneous absorption data, the experimental parameters in additional columns. HuskinDB was taken as a template, in order to facilitate the integration of these new data into the database. Some columns have also been added compared to HuskinDB (Data ID, Publication ID, CAS number, Category donor type, Category acceptor type). The publication ID is the number assigned to each publication during the systematic literature search. An error column has been added for the following parameters: Permeability coefficient K_p_, Steady-state Flux J_ss_, Maximum Flux J_max_, t_lag_. In these columns, we have reported the error as mentioned in the source publication. The value corresponds either to the standard error of the mean, or to the standard deviation; sometimes it is not specified.

The influence of different experimental parameters is discussed further in this publication, so that the user of the dataset can choose a set of data consistent with another one regardless of the type of analysis it may need (for instance, QSPR modeling).

This set of reliable and harmonizable human percutaneous absorption data has been designed to serve as a reference for aggregate exposure and risk assessment by federal and state governments, universities, and for research and development on transdermal drug delivery by the pharmaceutical and cosmetics industries. Our belief is that this dataset has the potential to uncover commonly utilized experimental conditions, which could then be recommended in future versions of international guidelines. By harmonizing practices and reducing result variability, these guidelines would promote consistency and reliability across experiments.

This dataset is well-suited for data extraction and its quality and richness are also assets for the development of robust *in silico* models^7^.

## Methods

**Fig. 4:**
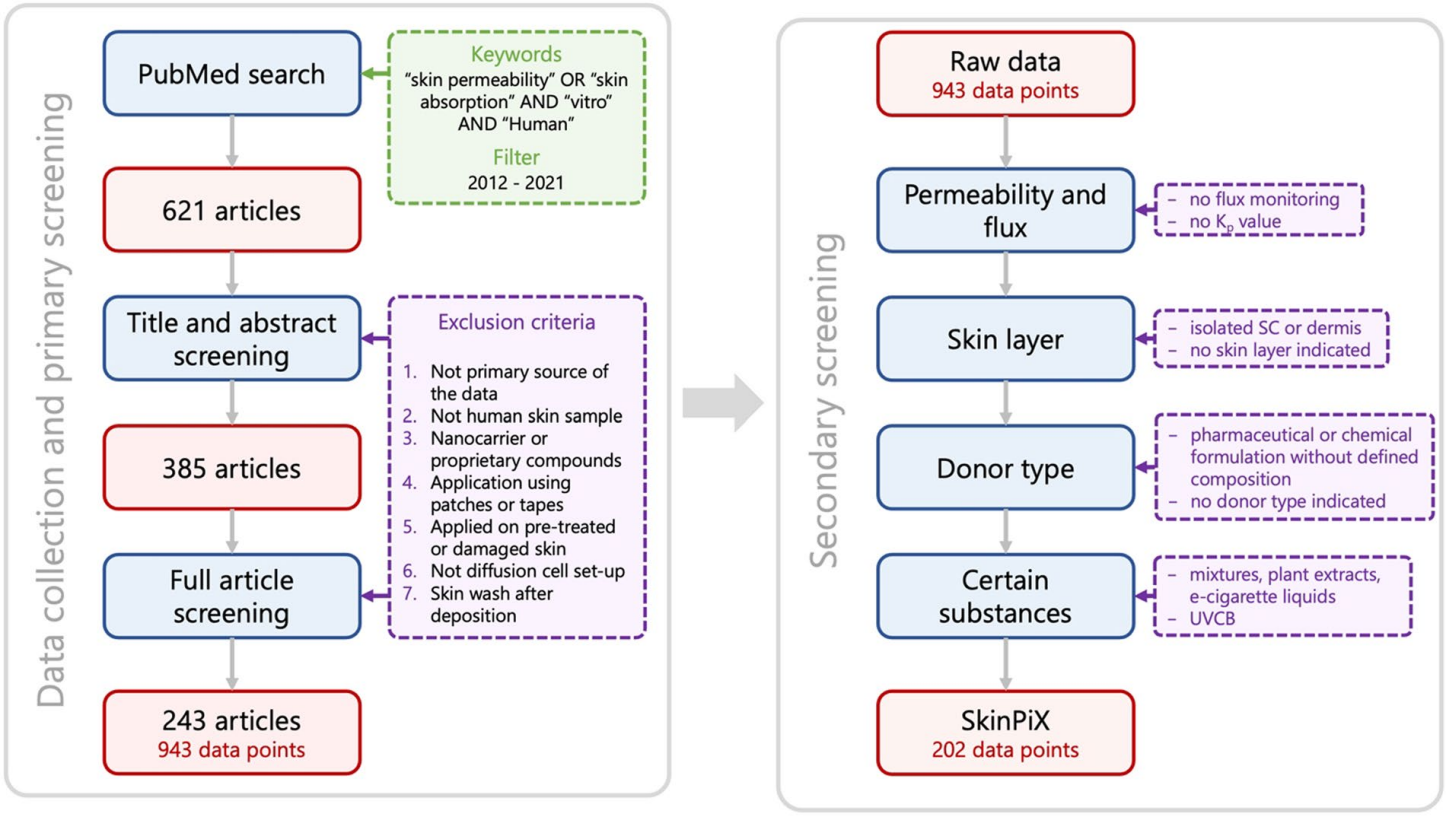
Data collection and filtering workflow. The process follows two main steps. First, relevant scientific publications were extracted using PubMed. Then skin permeability data were extracted along with relevant metadata. We kept only those data considered which met several criteria, as explained in section 1. “SC” stands for stratum corneum.

We conducted a systematic literature search and scrupulously analyzed the publications of interest to obtain a comprehensive dataset. The general workflow for creating the dataset is shown in Fig. 4. The aim was to cover as much as possible the new skin permeability data for well-defined organic compounds i.e. no UVCBs (unknown or variable composition, complex reaction products and biological materials) for instance, in an unambiguous experimental setup.

### Inclusion and exclusion criteria

We performed a systematic and comprehensive literature review of percutaneous absorption existing *in vitro* experimental data obtained between 2012 and 2021, using an automated approach. We searched publications in the major electronic database: PubMed, with a date restriction from January 2012 to June 2021 and the keywords “skin permeability” OR “skin absorption” AND “vitro” AND “Human”, resulting in 621 references in the public domain and the corresponding abstracts.

Considering the information in the abstracts, only publications in English and which were readily accessible were selected. The first manual sorting was performed and, according to the exclusion criteria for the analysis, we discarded publications:

1. without a primary source of the data (e.g., reviews, book chapters, datasets of published data or publications presenting predictive models, etc.) or already covered by the previous database HuskinDB (for 2012 publications);
2. with experiments conducted on animal, synthetic or artificial skin or Reconstructed Human Epidermis (RHE) and Skin (RHS) and Human Skin Equivalent (HSE) and other *in vitro* artificial skin models, cell lines and cultured skin, 3D organotypic constructs or experiments conducted *in vivo*;
3. with compounds formulated as nanocarriers, unidentified proprietary compounds;
4. with transdermal therapeutic systems (TTSs) of application of the substance, such as patches or tapes;
5. with percutaneous absorption experiments performed on pre-treated skin (laser, microneedles, etc.) or damaged skin;
6. without Franz cell or equivalent diffusion experimental set-up (microscopy, raman spectroscopy, microdialysis, transwell plate, etc.);
7. with percutaneous absorption results impacted by a skin wash after deposition.

In all cases where the abstract did not allow verifying these criteria, or in case of doubt, the reference was kept.

At this stage, 385 references were retained. The full article was obtained in PDF form for each of them. The articles were read and those that included experiments/data that did not meet the previous seven exclusion criteria were excluded.

Experiments not reporting the flux monitoring of substances deposited on the skin (only fraction absorbed or quantity measured in the different skin layers/distribution) were discarded. If no K_p_ was mentioned and if it was not possible to calculate it from pK_p_ or concentration and J_ss_, the reference was discarded.

Data obtained with isolated *stratum corneum* (SC) or isolated dermis alone were discarded so that only data from experiments carried out on the epidermis or epidermis + dermis were selected. Given the lack of clarity in the guidelines on the use of full thickness skin, we included full thickness skin experiment data in the dataset. If skin layer was not mentioned, the publication was discarded. Due to the unclear recommendations of the guidelines on the use of epidermal membranes separated by the heat separation method, data obtained with epidermal membranes were kept in the dataset.

Similarly, due to the inconsistent guidelines regarding the determination of mass-balance recovery, which is defined as the percentage of the original substance recovered at the end of an experiment, and the absence of systematic reporting of this recovery in literature, we decided not to exclude data for which the reported recovery was poor (<80% or >120%), but when available, the recovery mentioned in the publication was reported in the column notes of the dataset.

As indicated in the usage notes section, the occlusion of the donor compartment may impact the percutaneous absorption parameters. We chose not to exclude data obtained with occlusion but to mention it in the column notes of SkinPiX dataset.

We chose not to exclude any data on the basis of the acceptor type mentioned. The data obtained with the deposit of neat substances were reported and a specific work on donor types was carried out. If we look at the number of counts per donor type from different publications, no single donor type really stands out, with a maximum of only 11 publications using water. Data for which the composition of the donor medium was not provided were excluded. Formulations with UVCB, such as *MIGLYOL*^*®*^ *812 N, TWEEN*^*®*^ and *poloxamer 407*, were excluded. All pharmaceutical and chemical formulations of any kind were excluded because the donor type was poorly identified, or the precise composition was not known, or their production over time was not guaranteed, and/or the composition could vary over time. Some vehicles contain known enhancers but we chose not to remove them from the dataset. However, when a publication studied specifically the effects of enhancers, only the results of the substance deposited in a donor type without enhancers were kept.

We chose not to exclude any data based on skin storage temperature and storage duration since these parameters were not reported in 20% to 30% of the endpoints analyzed. However, when a compound was tested on both fresh and frozen skins, we chose to keep only the fresh skin data. Fresh skin maintains its original cellular structure, metabolic functions, and structural integrity, which are crucial elements influencing studies on permeability. These cannot be guaranteed after freezing and thawing processes. Thus, fresh skin data were given higher priority.

We chose to keep data regardless of the experimental temperature reported because this information was not mentioned in more than 50% of the data points (for acceptor medium temperature). In the dataset, the reported temperatures range from 32 °C to 37 °C, but it was not always clear whether they corresponded to the donor compartment, the skin or the acceptor compartment. If a publication reported data for multiple experimental temperatures (within the framework of the study of a temperature effect), only data collected in the experiments closest to 32 °C were kept.

Compounds meeting the definition of UVCB were excluded (compounds of vaping products and plant extracts). The parameters collected are indicated in the paragraph Data Records. Data processing was carried out using the KNIME Analytics Platform^18^. The KNIME workflows used to process the data are accessible in the online repository (10.57745/7FHQOY) for transparency. The resulting SkinPiX dataset contained 202 data points. The publications meeting all the inclusion criteria correspond to the 37 unique references^19–55^.

### Chemical space analysis of skin permeability data

The Generative Topographic Mapping (GTM) method^56^ was used to analyze the coverage of chemical space by HuskinDB and our new dataset. It is a dimensionality reduction method that transforms a multi-dimensional molecular descriptor space into a 2D latent space or a “map”^57^. This is accomplished by introducing a 2D manifold into the high-dimensional space and adjusting a normal probability density centered on it to fit the data distribution observed. Once the manifold is fitted, the compounds can be projected onto this 2D surface. The map can be colored based on population (density landscape) or property distribution (property/class landscape). The GTM class landscape was generated using ISIDA/GTM software.

## Data Records

The corresponding database version 1.1 is freely accessible at Recherche Data Gouv^58^.

The following information when available was collected and filled in an Excel sheet:

- Data ID and publication ID (integer): corresponds to the identifier given to each data entry and each unique publication. For a given publication ID, there can be several data ID with the same compound if there are percutaneous absorption experiments performed in different experimental conditions.
- SMILES (string): SMILES (Simplified Molecular Input Line Entry System) were extracted from the PubChem database, using the PubChem Identifier Exchange Service (https://pubchem.ncbi.nlm.nih.gov/idexchange/idexchange.cgi) by searching for molecules by their CAS (Chemical Abstracts Service) number.
- CAS number (string): unique and unambiguous CAS identifier that designates a specific substance. When not provided in the publication it was searched via PubChem (https://pubchem.ncbi.nlm.nih.gov/). CAS numbers of peptides are not defined.
- Compound name (string): for each substance, only one name or an amino acid sequence for peptide was entered in the dataset.
- K_p_ relation (string): signifies the exact K_p_ value (“=”) or if K_p_ value is smaller (“<”) or greater (“>”) than the value given in the K_p_ column.
- K_p_ and K_p_ error in cm•h^−1^ (float): the permeability coefficient (K_p_) value was obtained directly from the publication or calculated from the pK_p,_ (pK_p_ = - log K_p_) or calculated from J_ss_ and C_0_. The K_p_ (processed) column was used to harmonize K_p_ entries in decimal form. The same applied to log K_p_ (cm/s) (converted) column. When a range of K_p_ values has been reported in the publication, we have indicated the average K_p_ (processed) and log K_p_.
- Steady-state flux J_ss_ relation (string): signifies the exact J_ss_ value (“=”) or if the J_ss_ value is smaller (“<”) or greater (“>”) than the value given in the steady-state flux J_ss_ column.
- Steady-state flux J_ss_ and J_ss_ error (float) were first reported as written in the publication with their original unit. Then steady-state flux Jss (converted) and J_ss_ error (converted) in µg•cm^−2^•h^−1^ were also reported. These values were reported only if they were reported in the paper or if they could be calculated with the K_p_ and C_0_ given. If necessary, conversions were performed in *ad hoc* units.
- Maximum flux J_max_ and maximum flux J_max_ error in µg•cm^−2^•h^−1^ (float): J_max_ was reported when the substance was dosed pure or in its saturation concentration. As for J_ss,_ J_max_ and its error were first reported as written in the publication with their original unit and were then reported in µg•cm^−2^•h^−1^.
- t_lag_ and t_lag_ error in h (float): t_lag_ and t_lag_ error were reported when the data were available. The column t_lag_ (h) (processed) harmonizes entries.
- Skin donor type (string): the human skin used was either from a cadaver or corresponded to discarded surgical skin.
- Skin source site (string): the anatomical area was indicated (abdomen, breast, back, thigh).
- Skin preparation (string): it corresponds to the treatment carried out on the full thickness skin to obtain the skin used for the experiments. But very often, experiments implement split thickness skins which have been dermatomed. The layers of skin can also be separated (heat separation) providing epidermal membranes.
- Layer used (string): this section specifies which skin layer(s) was (were) used for the experiment: epidermis alone, epidermis and dermis. Sometimes the layer used was not explicitly indicated but when the skin was dermatomed with a possible indication of the thickness, we could deduce the layer used.
- Storage duration (days) (integer): when the skin was used fresh, this box was filled with “0”. In other cases, if the information was specified, then the storage duration was indicated in number of days or as a maximum number of days.
- Storage temperature (°C) (float): the skin was either used immediately or very quickly after collection (in this case, “used fresh” was indicated) or frozen or refrigerated before use. In these cases, the storage temperature was indicated.
- Donor type (string): indicated neat or diluted in a vehicle whose composition was given. Donor media were then classified into categories (column category donor type) (supplementary data 2).
- Donor pH (float): when provided, the pH value. If the experiments were carried out at different pH levels, only data relating to the pH levels most compatible with the skin were retained.
- Acceptor temperature (°C) and donor/skin surface temperature (°C) (float): the temperature was indicated if provided.
- Acceptor type (string): the composition of the acceptor medium was indicated. Acceptor media were then classified into categories (column category acceptor type) (supplementary data 1).
- Acceptor pH (float): when provided, the box was filled with the pH value.
- Cell type (string): type of permeation cell i.e. Franz diffusion cell (either static or flow through or modified Franz cell) or other type of diffusion cell. For Franz diffusion cells, if not specified in the publication, we have considered them to be static cells by default. If the publications did not explicitly mention that the experiments were carried out with Franz cells, we reported “other type of diffusion cell”.
- Author (string): first author’s name.
- Date of publication (integer): the year the article was published.
- DOI (string): DOI (Digital Object Identifier) is an unambiguous identifier of scientific publications.
- Notes (string): The experts have provided information on whether the experiment was carried out in occlusive or semi-occlusive conditions, whether the authors of the publication have stated the use of infinite dose conditions or if the K_p_ value was derived from a finite dose scenario. It also highlights the calculation of parameters and provides any other relevant information for the reader’s benefit such as mass balance recovery, insofar as it is available in the data source.

If the parameter of interest was not mentioned in the source publication, it was annotated “N/A”.

The different types of acceptors and donors were assigned to category labels for the analysis of the data set (supplementary data 1, 2).

## Technical Validation

The transcribed data were checked by a second reading by a researcher for accuracy and absence of error.

## Usage Notes

### Chemical space analysis of skin permeability data

**Fig. 5:**
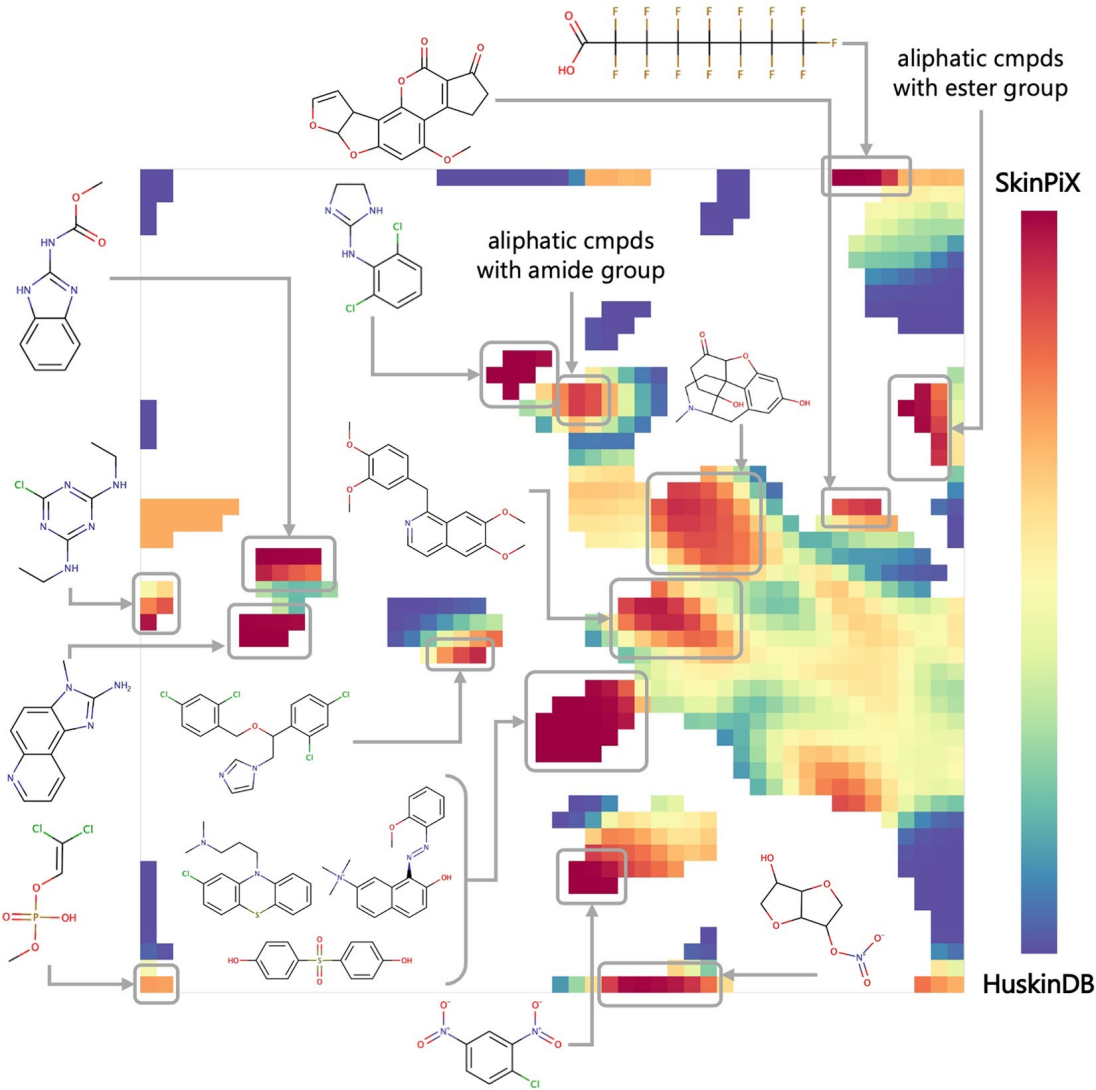
GTM landscape of skin permeability chemical space. Blue regions are mostly populated by compounds found in HuskinDB. Red regions are populated by compound data from SkinPiX. White regions do not contain any compound. The chemical content of various regions of the map is illustrated by example compounds (cmpds) and scaffolds.

In this work, we applied GTM to visualize the chemical space coverage of skin permeability data by using HuskinDB and SkinPiX (Fig. 5). The GTM class landscape shows that the main population of both datasets is located in the south-east quarter of the map (yellow, green and orange zones) while there are regions preferentially populated by HuskinDB (blue zones) or by SkinPiX (red zones). Examples of compounds and common chemical substructural features that are unique to SkinPiX (red zones) are indicated in Fig. 5. The *perfluorinated octanoic acid* and other short chain *fatty acids* (*4-chlorobutyric acid and 4-methylvaleric acid*) were added while the HuskinDB originally contained 2 long chain instances of this chemical family *(linolenic acid and oleic acid*). The addition of *dinitrochlorobenzene* completed the Structure Activity Relationship of *nitrobenzene* compounds. We have also added 3 *benzimidazoles* and 2 *isosorbides*, as these molecular scaffolds were not present in the original Huskin database. *Bisphenols*, *chlorpromazine* and *basic red 76* (as well as other compounds containing *dibenzo-dinitrogens*) were also absent from the original HuskinDB. However, the large part of SkinPiX covers the same region as HuskinDB: they are represented in the map as green to orange regions that are also the most densely populated. The GTM analysis showed that SkinPiX expands the chemical space of skin permeability by introducing new molecular scaffolds.

### Factors influencing *in vitro* skin permeability

One should be aware that the percutaneous absorption results of a given substance (K_p_, J_ss_) are influenced by many factors such as donor composition, acceptor composition and other experimental conditions^12,15,16,59^.

Hence, when seeking to find the K_p_ or J_ss_ values for a particular substance from SkinPiX, it is beneficial to examine the experimental parameters that were utilized to obtain those results. Consulting the following section may help the user in determining whether the results for a given substance under different experimental conditions are comparable or not.

- Skin donor type
  The guidelines proposed to carry out the studies either with human skin from autopsies (cadaver skin) or with surgical discard skin, the two main sources of supply^10^. Surgical discard skin is generally preferred but cadaver skin, which can be more convenient, is also accepted for percutaneous absorption studies as long as the integrity of the barrier is verified^16^, as the decrease of barrier integrity could lead to increased permeability. Nevertheless, as the skin is not considered viable, the metabolism of the substance cannot be studied. It is good practice to ensure before using cadaver skin that the skin does not metabolize the substance studied or that its metabolism does not have an impact on the flux. It is important to know how long and how the skin was kept before the experiment^16^. However, as the conditions in which cadaver skin is kept are variable, and the decomposition of the different components of post-mortem skin is a complex process, surgical samples are often preferred and recommended^17^. Although the gender, age and phenotype of the skin donor may also have an impact on the percutaneous absorption of a substance^60^, they were not taken into account in SkinPiX.
- Skin source site
  Bormann *et al*., reviewed the impact of anatomical location on percutaneous penetration in humans *in vivo* with greater penetration on the face, neck and genital area^61^. The differences observed *in vivo* can be explained by, among other things, the thickness of the SC, the density and size of hair follicles, hydration and the extent of blood irrigation^12,60,62^. With *in vitro* percutaneous absorption experiments, a similar variability and the same trends have also been observed according to body zones^63,64^.
  The forearms and the hands are generally the most exposed cutaneous regions during occupational exposure to chemicals, but in practice most dermal absorption experiments use skin from abdomen or breast/chest skin samples obtained from aesthetic surgery, as mentioned in GN156 and GD28^8,11^. Note that results sometimes include experiments performed on skins from several anatomical areas.
- The layer used
  It is important to consider which layer of the skin was used to obtain experimental data when analyzing skin permeation values, as the different layers do not have the same permeability properties. Thinner skin thickness generally leads to a higher flow rate. But since the dermis is a predominantly hydrophilic tissue, its presence (in the case of dermatomed skin) or its absence (epidermis alone) has a greater impact on the percutaneous absorption of lipophilic substances^65^. The latest version of GN156^11^ recommends the use of split thickness skin of 200 to 400 µm which includes the SC, the viable epidermis and part of the dermis. The use of the viable epidermis and dermis in addition to the SC ensures better representation of the *in vivo* skin structure of the skin layers insofar as the viable epidermis and dermis can also have an impact on the diffusion of a chemical through the skin^66^. Although they were proposed in GD28^8^ and TG428^9^ dating from 2004, and in the 2019 version of GN156^10^, epidermal membranes (SC + viable epidermis) obtained from heat separation no longer appear to be recommended in the latest GN156 version of 2022^11^ insofar as they could, due to their insufficient barrier function, lead to overestimating the absorption results compared to dermatomed skin. The use of isolated SC presents a big disadvantage: this layer lower than 0.1 mm is very fragile and it can be tricky to work on unaltered membranes with an intact barrier^67^. Note that there is no mention of the possible use of isolated SC in the current OECD guidelines.
  It is clearly stated in the 2019 version of GN156 that full thickness skin cannot be used to determine flux^10^, certainly because the penetration of lipophilic substances is greatly reduced with full thickness skin compared to split thickness skin^68^. Surprisingly this information is not reported in the latest version of the guidance note^11^.
- Skin preparation
  In addition to skin used without preparation (full thickness skin without fat), either the skin is dermatomed to obtain split thickness skin of controlled thickness (see previous paragraph), or the epidermis is separated from the dermis. There are several epidermis-dermis separation methods^69^, the most commonly used being heat separation. Epidermal membranes and dermatomed skin are both accepted even if, according to the 2019 version of GN156^10^, dermatomed skin is the most appropriate model. However, care must be taken to ensure that the heat separation technique does not alter the permeation properties of the skin. The method of skin preparation might have an impact on skin enzymes present in skin and can impact the results. In the case of esters, for example, the relevance of the data obtained with the epidermis is a subject of discussion as Lau *et al*., have shown that the heat separation technique could significantly decrease the activity of *esterases*^70^.
- Skin storage temperature
  For percutaneous absorption experiments, skin is generally used immediately after excision or at least within 24 h (fresh skin) or stored frozen for up to several months according to GD28^8^. However, Dennerlein *et al*., questioning the validity of the experiments on which the guidance document is based, carried out experiments showing that up to 30 days of freezing at −20 °C did not significantly alter the permeability of skin with respect to the 3 substances tested compared to freshly excised human skin^71^. Jacques-Jamin *et al*., came to the same conclusion with 3 other substances and slightly longer freezing times of 8 and 12 weeks^72^. On the other hand, storage at −80 °C may increase permeability and is not recommended^8,73^. For practical reasons, it is best to remove the subcutaneous tissue before freezing the skin. Repeated freezing and thawing are not recommended as this can damage the barrier. Frozen skin should not be used for substances metabolized by the skin, as the activity of enzymes may be altered and inactivated by freezing. Because the effect of freezing on the percutaneous absorption parameters of skin may depend on several factors, such as how the skin is frozen (full thickness, dermatomed, epidermal membranes), it is necessary to check the integrity of the barrier after storage in the freezer according to GD28^8^.
- Storage duration
  The TG428 recommends using fresh skin within 24 hours after excision^9^. Based on recent publications, if skin is stored frozen at −20 °C, it must be kept for short periods of 1 to 3 months to obtain accurate and reliable permeation parameters^71,72^.
- Cell type
  Static, with appropriate continuous stirring of the acceptor fluid^74^, and flow-through diffusion cells are both acceptable for skin *in vitro* absorption experiments according to all the OECD guidelines, insofar as they are composed of inert material^8,9,11^. Some authors have summarized the advantages and drawbacks of each system^17,75^.
  Studies have shown similar results for these two types of cells^76,77^. In the framework of their comparison study, Van de Sandt *et al*., concluded that the type of cell and its design have little impact on the results^65^. The terms used to describe the cells used experimentally vary according to the authors, which does not always make it possible for them to be classified precisely.
- Experiment temperature
  Numerous studies and GN156 indicate that experiment temperature is a crucial parameter to control as it affects the passive diffusion of substances and therefore their flux and lag time^11,78–80^. That is why TG428 and GD28 recommend keeping skin and the diffusion cell, in particular the acceptor chamber, at the physiological temperature of human skin, i.e. 32 ± 1 °C^8,9^.
- Acceptor type
  The type of acceptor is very important in *in vitro* percutaneous absorption experiments^16^. All the guidelines for *in vitro* dermal absorption testing agree that the type of acceptor used must not be a limiting factor in the permeation process^81^. The solubility of the substance in the medium must be at least 10 times the maximum expected concentration (GN156)^11^. The acceptor fluid should not affect skin integrity^81^. GN156 proposes using a normal saline for hydrophilic substances and non-viable skin^11^. For lipophilic compounds, GN156 indicates “the acceptor fluid may contain solvent mixtures such as *ethanol* and *water* (50% *aqueous ethanol*), < 6% *polyoxyethylene oleyl ether* in water, or 5% *bovine serum albumin*”^11^. However, in order to maintain viable skin, the acceptor should preferably be physiologically compatible with the skin (GD428 and GD28), such as a tissue culture medium, in particular to consider metabolism^8,9^. An acceptor fluid with a high buffering capacity is required to guarantee the viability of the skin throughout the experiment. It is advisable to add glucose and antibiotics to the acceptor fluid to prevent the skin from deteriorating, especially for experiments lasting more than 24 hours^82^. Its precise composition must be indicated.
  Since the acceptor fluid has a major effect on skin absorption parameters, the guidelines should be more precise on this subject, as requested by a group of experts in the field^15^, and should propose for each situation precise compositions of the acceptor fluids, which would ensure that the future K_p_, J_ss_ and J_max_ data found in the literature are not impacted by this parameter.
  Figure 5 shows the acceptor category types included in the dataset.
- Acceptor pH
  Only GD28 gives information on the pH of the acceptor medium: “for non-viable skin preparations, the acceptor fluids for evaluating water soluble compounds are usually saline solutions, pH 7.4”, which correspond to quite specific conditions^8^. The pH must take into account the more general recommendations on the acceptor type: it must not affect the integrity of the barrier, and adequate solubility of the test substance in the acceptor fluid should be demonstrated (TG 428)^9^. As a general rule, the acceptor fluid is aqueous. Wagner *et al*., investigated the impact of the pH of the acceptor fluid (pH buffer 5.5, 7.4, 8.5 and 9) on the pH of the different skin layers^83^. After reaching an equilibrium of 3 h with the medium, the pH of the dermis and the viable epidermis is modified, becoming close to that of the medium. A change in the pH of the skin can affect the permeation of the test substance in several ways. To maintain the viability of skin explants, some authors advise using a survival medium with a high buffering capacity to maintain a physiological pH above 5.5 for the duration of the experiment to compensate for the production of lactate by the skin (otherwise the medium must be renewed regularly)^82^. Hopf *et al*., even recommended a pH close to 7.35^15^.
- Donor type
  The influence of the formulation or vehicle on skin penetration is evident and well documented, as certain vehicles or vehicle components help test substances to cross the SC barrier^84,85^, modify the flux and the t_lag_^13,55,86^. This is why GN156 recommends that the test preparations are similar to what humans are exposed to^11^. But as exposure situations vary, the dataset includes innumerable donor types whose effects on the percutaneous absorption of the substance tested are different.
  Measurements extracted from permeation experiments should be compared with experiments conducted on identical vehicles since K_p_ is a parameter that incorporates the partitioning step of the compound between the vehicle and the SC layer of the skin. A vehicle of interest in this perspective could be water but we must keep in mind that water modifies some skin properties (hydration, swelling, etc.)^13^. Moreover, this raises the question of substances that are not very soluble in water, such as lipophilic substances. Below a certain solubility in water, a consensus-based vehicle other than water should be proposed.
  Figure 5 illustrates donor category types included in the dataset.
- Donor pH
  GN156 warns about the potential pH effects of the formulation: it can modify the ionization state of the substance tested and have deleterious effects on the skin: irritation resulting in modifying the skin’s absorption parameters^11^. However, no pH value is recommended.
  The question of a donor pH is only relevant for aqueous formulations. The physiological surface pH of skin is acidic, around 5, and there is a pH gradient across the thickness of the SC^87^, with some publications indicating a gender dependence of skin pH^83,88,89^. The pH of *in vitro* SC (frozen or fresh) is higher and can become neutral^83,90^. The deposition solution is generally at a pH between 4 and 7, taking into account the buffering capacity of the skin^91^. Caution is required as even in this range very different fluxes can be observed^92,93^.
  The buffering capacity of the skin is limited and can be overcome in case of exposure to solutions with extreme pH, as they can modify the skin barrier^87^.
  Knowing that the ionized forms of a substance are much less permeable than the non-ionized forms, the pH of the donor medium will necessarily have an effect on the parameters in the case of ionizable substances at non-extreme pH, as observed for example for lignocaine flux^94^.
- Occlusion
  According to GN156 the choice of occlusion/non-occlusion should depend primarily on the properties of the test substance (occlusion to prevent the evaporation of volatile substances) and the exposure scenario^11^. Generally, but not always, occlusion favors the percutaneous absorption of the test substance by increasing skin (SC) hydration and temperature, leading to a modification of the percutaneous absorption parameters^95^. Bjorklund *et al*., showed that by decreasing the water gradient over the skin and thus increasing its hydration, the flux of 2 substances, one hydrophilic, the other lipophilic, increases drastically^96^. These results help to explain the effects of occlusion. Van der Merwe *et al*., observed the impact of occlusion on the apparent lag time^80^.
- Finite-infinite dosing scenario
  The K_p_ is calculated from the flux of the solute over the skin under steady-state conditions, i.e. in infinite dosing conditions. Indeed, steady-state is rarely reached in finite dose conditions. TG428 advise to apply up to 10 µl/cm² in finite dose experiments on liquids and 100 µl/cm² or more in infinite dose experiments^9^. However, it is necessary to consider that these recommendations have certain limits, for example, a small volume of highly concentrated solution of a low permeated solute can behave like an infinite dose scenario^97^. Therefore, a better mathematical definition is that finite dose conditions apply when depletion of the donor occurs^98^ with the characteristic curve shapes presented in Fig. 2. Unfortunately, in practice, some researchers claim they are in infinite dose conditions but only give the deposited volume used. Several authors report a J_ss_, K_p_, and t_lag_ without mentioning whether they had previously verified that they obtained a steady-state and how they verified it.
  TG428, GD28 and GN156 do not comprehensively address methodological issues to determine the boundaries of the steady-state and the K_p_ in infinite dose, nor do they indicate if it possible to predict a K_p_ without steady-state, nor do they propose any criterion to evaluate the quality of the K_p_ obtained^8,9,11^.
  It is possible to extrapolate K_p_ from finite dose experiments but the estimated K_p_ are generally lower than the true values^15^.
- Reaching steady-state
  The methodology used to determine steady-state boundaries has a significant impact on the percutaneous absorption parameters as the inclusion of data collected at times before steady-state leads to underestimating both K_p_ and t_lag_^99^. The time recommended for the permeation rate across a membrane to reach the steady-state value must be at least 2.7 or 3 times the lag time in order to obtain a good estimate of the permeability coefficient^98,100,101^. Niedorf *et al*., proposed an automated approach based on an algorithm to define the boundaries of the steady-state^102^.
- Mass-balance recovery
  At the end of the experiment, mass-balance recovery must be determined and provided (TG 428)^9^. The GD28 and GN156 set an adequate recovery target for the test substance of 90 to 110% with a recovery of 80 to 120% tolerated for volatile and non-radiolabeled substances^8,11^. In the case of recoveries outside this range or for non-indicated recoveries, the results obtained are questionable. Indeed, an excessively weak recovery can be due, for example, to the evaporation or adsorption of substances, particularly for lipophilic ones, on the walls of the vials or donor/acceptor compartments, or a problem of the extraction of the test substance from the skin^15,17^. However, GD28 indicates “For infinite dose applications, a steady-state flux and a permeability coefficient (K_p_) are determined. Recovery determination is not relevant because the only important end-point is the appearance of the test substance in the acceptor fluid”^8^.

This work highlights the serious need for standardization and exhaustive and comprehensive reporting of experimental conditions in skin absorption studies. Future corrections or updates of the SkinPiX dataset will lead to version increments and new DOIs referring to these new versions. The dataset has been designed in order to facilitate its integration in the HuskinDB. We think that it is more useful to contribute to an existing database, in a formal or informal way, rather than creating a new one on the same subject. An enhancement to this dataset could be to add flux data collected from finite dose scenarios, offering potential value to the toxicokinetics and modelling community. This dataset is limited to J_ss_ / J_max_ and K_p_, data determined from substance concentrations in the receptor fluid. A comprehensive dataset of the distribution of the permeant in the various compartments is not presented. Inclusion of such data could bring added value to fit and evaluate *in silico* models of skin absorption.

The data descriptor was peer reviewed in 2024 based on the version 1.1 of the dataset available on the platform *Recherche Data Gouv*^58^ at that time.

### Supplementary information

Supplementary Information: An update of skin permeability data based on a systematic review of recent research

## Data Availability

The generated dataset (SkinPiX)^58^ version 1.1 is available in open source (10.57745/7FHQOY) and the KNIME workflows used to process the data are provided there.
