# Supplementary material for: An update of skin permeability data based on a systematic review of recent research
Source: Sci Data. 2024 Feb 21;11:224. doi: 10.1038/s41597-024-03026-4 (PMC10881585; doi:10.1038/s41597-024-03026-4)
Supplement: Supplementary file 1 — Supplementary Information: An update of skin permeability data based on a systematic review of recent research [file 41597_2024_3026_MOESM1_ESM.pdf]

Supplementary Information: An update of skin permeability data based on a systematic review of recent research

Contents

Supplementary data 1. Acceptor types and corresponding category labels 2
Supplementary data 2. Donor types and corresponding category labels 3

Supplementary data 1. Acceptor types and corresponding category labels

The following table lists the different types of acceptor and the category labels to which they have been assigned. Acceptor types are separated using ";" as a delimiter.

Acceptor type category	Acceptor type (original)
PBS based formulations	PBS; PBS with BSA; Isotonic phosphate buffer solution containing 20% PEG 400; PBS with sodium azide; PBS containing 10 mM phosphate buffer 137 mM NaCl and 2.7 mM KCl at pH 7.4 with 0.005% of gentamicin sulfate as a preservative; PBS 10 mM; PBS containing 10% ethanol; Diisopropyl fluorophosphate in PBS at a concentration of 2.7 µM/mL; PBS aqueous solution 0.01 M containing 6% v/v polyethylene glycol; Dulbecco's phosphate buffered saline with calcium and magnesium + 2% BSA; Propylene glycol/PBS 2:3 v/v; PBS and 0.02% NaN3
NaCl solution	Physiological solution NaCl 0.9%; NaCl solution; 0.9% NaCl, 5% bovine serum albumine, 0.1% hexamycine in water
Cell culture media	DMEM based culture medium and 5% BSA; Hanks balanced salt solution with 50 mg/L of gentamicin sulfate, 5.96 g/L of HEPES; RPMI 1640 with gentamycine, penicillin, streptomycin and 2% BSA; Minimum essential medium eagle, supplemented with 2% w/v BSA; Hanks balanced salt solution with 50 mg/L of gentamicin sulfate, 0.32 g/L of sodium bicarbonate, 5.96 g/L of HEPES
Waterbased solution	5% BSA in water

Table S1. Acceptor types and corresponding category labels

Supplementary data 2. Donor types and corresponding category labels

The following table lists the different types of acceptor and the category labels to which they have been assigned. Acceptor types are separated using ";" as a delimiter.

Donor type category	Donor type (original)
PBS based formulations	PBS; PBS and 10% ethanol; Dulbecco phosphate buffered saline with calcium and magnesium with sodium azide at 0.5 mg per mL with antioxidants 0.3% ascorbic acid and 0.4% sodium sulfite; Dulbecco phosphate buffered saline with calcium and magnesium with sodium azide at 0.5 mg per mL; Dulbecco phosphate buffered saline with calcium and magnesium with sodium azide at 0.5 mg per mL with 0.056% toluene; PBS and 0.02% NaN3
Artificial sebum	Artificial sebum 15 % squalene, 25 % jojoba oil, 30 % glycerol trioleate, and 30 % oleic acid
Other alcohols and associated mixtures	Isopropyl alcohol
Glycols and associated mixtures	Propylene glycol; 60% propylene glycol; Propylene glycol and 5% n-methyl-2-pyrrolidone; Propylene glycol and 5% azone; Propylene glycol and 5% ethanol; Propylene glycol and 5% oleic acid; Propylene glycol and 5% transcutol P; Propylene glycol/water 1:1; Propylene glycol/propylene glycol monolaurate 1:1 v/v; Propylene glycol/water 6:4 v/v
Other buffer	0.1 M citrate buffer; Citrate buffered saline; Carbonate buffered saline; Modified Krebs Henseleit buffer
Water	Water; Waterbased solution
Ethanol and ethanol based formulations	Ethanol/water 7:3 v/v; Ethanol; Ethanol and 5% n-methyl-2-pyrrolidone; Ethanol and 5% azone; Ethanol and 5% oleic acid; Ethanol/water 1:1 v/v; 45% ethanol in water; Ethanol/propylene glycol 7:3 v/v; Ethanol with 10% propylene glycol; Ethanol with 10% isopropyl myristate; Ethanol with 10% octyl salicylate
Others	Isopropyl myristate; Dimethylamine salt solution with EDTA and silicone in water; Dimethyl isosorbide; n-Methyl-2-pyrrolidone

Table S2. Donor types and corresponding category labels
